# Ongoing monitoring of data clustering in multicenter studies

**DOI:** 10.1186/1471-2288-12-29

**Published:** 2012-03-13

**Authors:** Lauren B Guthrie, Emily Oken, Jonathan AC Sterne, Matthew W Gillman, Rita Patel, Konstantin Vilchuck, Natalia Bogdanovich, Michael S Kramer, Richard M Martin

**Affiliations:** 1 Obesity Prevention Program, Department of Population Medicine, Harvard Medical School and the Harvard Pilgrim Health Care Institute, Boston, USA; 2 School of Social and Community Medicine, University of Bristol, Bristol, UK; 3 Department of Pediatrics, McGill University Faculty of Medicine, Montreal, Canada; 4 Department of Epidemiology and Biostatistics, McGill University Faculty of Medicine, Montreal, Canada; 5 The National Research and Applied Medicine Mother and Child Center, Minsk, Republic of Belarus; 6 Obesity Prevention Program, Department of Population Medicine, Harvard Medical School and Harvard Pilgrim Health Care Institute, 133 Brookline Avenue, 6th Floor, Boston, MA 02215, USA

## Abstract

**Background:**

Multicenter study designs have several advantages, but the possibility of non-random measurement error resulting from procedural differences between the centers is a special concern. While it is possible to address and correct for some measurement error through statistical analysis, proactive data monitoring is essential to ensure high-quality data collection.

**Methods:**

In this article, we describe quality assurance efforts aimed at reducing the effect of measurement error in a recent follow-up of a large cluster-randomized controlled trial through periodic evaluation of intraclass correlation coefficients (ICCs) for continuous measurements. An ICC of 0 indicates the variance in the data is not due to variation between the centers, and thus the data are not clustered by center.

**Results:**

Through our review of early data downloads, we identified several outcomes (including sitting height, waist circumference, and systolic blood pressure) with higher than expected ICC values. Further investigation revealed variations in the procedures used by pediatricians to measure these outcomes. We addressed these procedural inconsistencies through written clarification of the protocol and refresher training workshops with the pediatricians. Further data monitoring at subsequent downloads showed that these efforts had a beneficial effect on data quality (sitting height ICC decreased from 0.92 to 0.03, waist circumference from 0.10 to 0.07, and systolic blood pressure from 0.16 to 0.12).

**Conclusions:**

We describe a simple but formal mechanism for identifying ongoing problems during data collection. The calculation of the ICC can easily be programmed and the mechanism has wide applicability, not just to cluster randomized controlled trials but to any study with multiple centers or with multiple observers.

## Background

Multicenter study designs have several advantages, including the opportunity for larger sample sizes and greater generalizability of findings than single-site studies. Along with these advantages, however, comes the possibility of inter-site variability as a result of procedural differences between the study centers, such as variations in applying the study protocol [1]. Systematic variation between centers will result in a high degree of clustering, whereby measurements on study subjects from the same site are more highly correlated with each other than with measurements from different sites. Clustering caused by such systematic measurement error can result in bias and, in multicenter studies, will substantially reduce study power. Systematic measurement error is especially concerning in cluster-randomized trials, a type of multicenter study in which an intervention is delivered to an entire group, especially when measurements are clustered within the same groupings that serve as the units for cluster randomization [2].

Although these problems can be assessed at the stage of statistical analysis [1,3], it is preferable to identify and rectify them during data collection. Quality assurance efforts aim to reduce the magnitude of random and systematic errors, thereby enhancing the reliability and validity of the data [4]. In general, quality assurance plans should include actions that aim to prevent errors and to detect and reduce those that occur [4]. The study's size and design will dictate the appropriate quality assurance procedures to use. Large multicenter studies, for example, often require extensive data monitoring efforts organized by a centralized group [5].

In this article, we describe quality assurance efforts aimed at reducing cluster-associated measurement error in a recent follow-up of a large cluster-randomized controlled trial. We describe periodic assessment of intraclass correlation coefficients (ICCs). Although we use a cluster-randomized trial as an example, such an approach can be applied to the ongoing monitoring of data collection in all multicenter studies.

## Methods

### Quantifying clustering--The ICC

The ICC is a statistic that can be used to quantify the degree to which observations within a cluster differ from those between clusters. A summary of the theory of and methodology for ICC calculation can be found in a paper by Donner [6]. We calculated confidence limits for the ICC's using the multivariate delta method [7].

When employed in a multicenter study, the ICC compares the variance between centers (${\text{σ}}_{\text{b}}^{\text{2}}$) to the total variance (i.e. the variance within centers (${\text{σ}}_{\text{w}}^{\text{2}}$) plus the variance between centers (${\text{σ}}_{\text{b}}^{\text{2}}$)), where the $\text{ICC}=\sigma_{\text{b}}^{\text{2}}/\left(\sigma_{\text{b}}^{\text{2}}+\sigma_{\text{w}}^{\text{2}}\right)$[8]. If all the variation is between centers (i.e. ${\text{σ}}_{\text{w}}^{\text{2}}=0$), then the ICC = 1. If all the variation occurs within centers (${\text{σ}}_{\text{b}}^{\text{2}}=0$), then the ICC = 0.

The ICC is frequently used to assess the degree of clustering in multicenter studies during data analysis, after data collection is complete. However, we believe that it can also be used as a component of periodic monitoring during data collection. Suppose the centers are in the process of measuring a specific physical characteristic of a homogenous population. A high ICC would indicate higher than expected variation due to clustered measurement between centers. If detected early, the reasons for the clustered measurement may be corrected.

### The promotion of breastfeeding intervention trial (PROBIT)

To illustrate how the ongoing calculation of ICCs can be used to monitor and reduce measurement error in multicenter studies, we review our recent experience with the Promotion of Breastfeeding Intervention Trial (PROBIT). Thirty-one maternity hospitals in the Republic of Belarus, and one associated outpatient polyclinic for each hospital, were randomly assigned to implement a breastfeeding promotion intervention or to continue the procedures in place at the time of randomization; 17 046 mother-infant pairs were recruited in 1996/1997 [9,10]. We include here data from the most recent follow-up in 2008-2010, when the children were aged 11.5 years. Children were seen by 39 pediatricians at the 31 polyclinics, herein labeled alphabetically from A to AE. PROBIT was registered with Current Controlled Trials as ISRCTN37687716.

In September 2007, we held a weeklong workshop to train pediatricians to perform the measurements to be collected in PROBIT children at age 11.5 years. Training included lectures and practical sessions. We also gave each pediatrician a manual of procedures and instructive DVD in Russian. Study visits commenced in January 2008. We held a re-training workshop in March 2008 and biannually thereafter. At the visits, pediatricians measured child height (using stadiometers with moveable headboards); circumferences (using non-stretchable measuring tapes); skinfold thicknesses (using Lange spring-loaded calipers); weight and bioelectrical impedance (using the TANITA TBF 300 GS body fat analyser); blood pressure (using OMRON 705IT devices and appropriately sized cuffs); and fasting glucose (using Roche Advantage Accu-Chek Glucometers). At the biannual re- training workshops we monitored follow-up rates, clarified procedures, and re-standardized measurements. Lastly, independent study monitors visited each clinic early in the data collection process for on-site monitoring visits, to ensure early correction of faulty/inconsistent techniques.

The study received ethical approval from McGill University Health Center Research Ethics Board; the Human Subjects Committees at Harvard Pilgrim Health Care; and the Avon Longitudinal Study of Parents and Children Law and Ethics Committee. Prior to any measurements being taken, a parent or legal guardian provided written informed consent and all children provided written assent. PROBIT has been registered as ISRCTN-37687716 and conforms to the CONSORT recommendations for the design, analysis, and reporting of cluster-randomized trials [11].

We received periodic data downloads from the data coordinating center in Minsk, approximately every three months. Upon receipt, we checked for implausible values or extreme outliers that might represent transcription or data entry errors. We reported all outliers to the data entry technicians, who corrected the database by comparing against the paper records, or in consultation with the pediatricians. We calculated ICCs on all continuous measurements using SAS version 9.2 (Cary, NC).

## Results

The left-hand column of Table 1 shows the ICC calculations (with 95% confidence intervals) from the first data download in September 2008, involving 1,572 children from 15 polyclinics. The ICCs for standing height, weight, mid-upper arm circumference, hip circumference, head circumference, triceps and subscapular skinfold thickness and glucose were all relatively low (< 0.10), indicating modest clustering within polyclinics. The ICC of 0.005 for impedance indicated that this measurement was particularly consistent across polyclinics--perhaps not surprising, as impedance is measured using an automated scale, and is therefore less subject to differences in measurement technique.

**Table 1:** Intraclass correlation coefficient (ICC) calculations (with 95% confidence intervals) for continuous outcome measurements at data downloads between September 2008 and May 2010

Measurement	ICCs (95% CI) by date of data download			
	**9/08**	**3/09**	**10/09**	**05/10**
	
	**n = 1,572** **15 clinics**	**n = 4,865** **27 clinics**	**n = 8,949** **31 clinics**	**n = 12 374** **31 clinics**

Standing height	0.06 (0.01, 0.11)	0.03 (0.01, 0.06)	0.03 (0.01, 0.05)	0.03 (0.02, 0.05)

Sitting height	0.92 (0.86, 0.97)	0.28 (0.17, 0.39)	0.03 (0.01, 0.04)	0.03 (0.01, 0.05)

Weight	0.03 (0.00, 0.06)	0.02 (0.00, 0.03)	0.02 (0.01, 0.03)	0.02 (0.01, 0.03)

Bioimpedance	0.005 (0.00, 0.01)	0.004 (0.00, 0.01)	0.01 (0.00, 0.01)	0.01 (0.00, 0.02)

Mid-upper arm circumference	0.04 (0.00, 0.08)	0.02 (0.01, 0.04)	0.03 (0.01, 0.05)	0.03 (0.01, 0.05)

Systolic blood pressure	0.16 (0.05, 0.26)	0.11 (0.05, 0.17)	0.12 (0.06, 0.17)	0.12 (0.06, 0.17)

Waist circumference	0.10 (0.02, 0.18)	0.10 (0.05, 0.15)	0.09 (0.05, 0.14)	0.07 (0.04, 0.11)

Hip circumference	0.05 (0.00, 0.10)	0.04 (0.01, 0.06)	0.03 (0.01, 0.05)	0.03 (0.01, 0.05)

Head circumference	0.02 (0.00, 0.05)	0.05 (0.02, 0.08)	0.13 (0.07, 0.19)	0.10 (0.06, 0.15)

Triceps skinfold thickness	0.09 (0.02, 0.16)	0.07 (0.03, 0.11)	0.11 (0.06, 0.17)	0.12 (0.07, 0.18)

Subscapular skinfold thickness	0.07 (0.01, 0.13)	0.04 (0.02, 0.07)	0.04 (0.02, 0.06)	0.04 (0.02, 0.06)

Glucose	0.05 (0.01, 0.09)	0.08 (0.03, 0.13)	0.09 (0.04, 0.13)	0.08 (0.04, 0.11)

**Figure 1:**
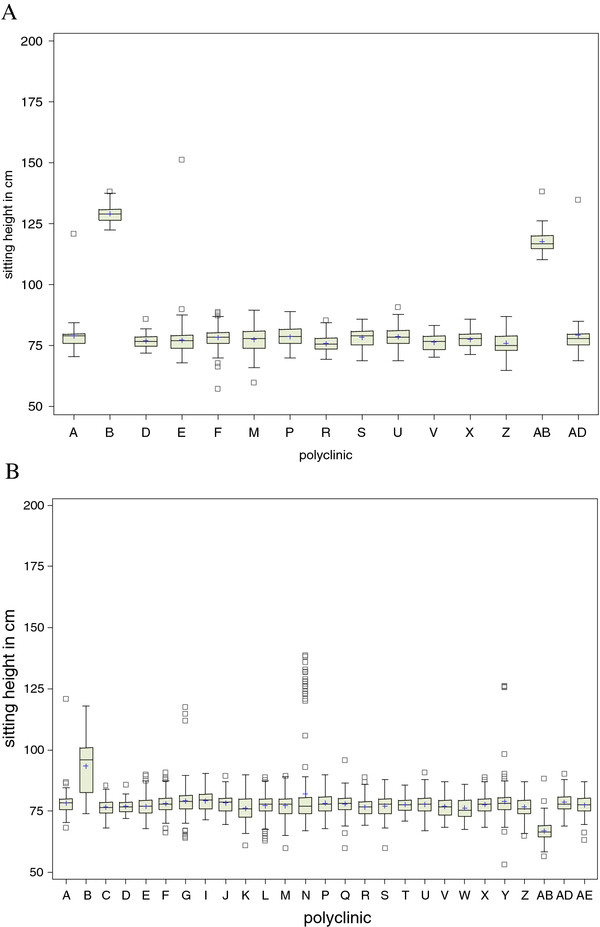
**Side-by-side box-plot of sitting height (cm) measurements, by polyclinic**. (Missing polyclinics in each box-plot reflect polyclinics that had either not commenced recruitment, or had no records entered into the database at the date of the download). 1A: September 2008 data download (total n = 1,572 records from 15 polyclinics). 1B: March 2009 data download (total n = 4,865 records from 27 polyclinics)

The ICC of 0.92 for sitting height, however, was higher than expected based on a previous follow-up of PROBIT at age 6.5 years (when the ICC was 0.09) [10]. To identify the reason for such a high degree of within-center clustering, we constructed side-by-side box-plots of sitting height at each polyclinic (Figure 1a). The plot revealed that the mean sitting height measurements of polyclinics B and AB were approximately 50 cm higher than the other clinics.

We suspected that these two clinics had been using an incorrect ruler to obtain the sitting height measurement. At the start of the study, we provided each pediatrician with a standard wall-mounted stadiometer containing two integrated rulers: one for measuring standing height and one for sitting height. The sitting height ruler started at 50 cm above the floor, to account for the standard 50 cm study stool upon which the child was seated while measured. Pediatricians at these two polyclinics confirmed that they had been using the wrong ruler, and we adjusted the measurements by 50 cm.

Results from a subsequent download six months later (4,865 records from 27 polyclinics) revealed that the ICC for sitting height had dropped markedly (from 0.92 to 0.28); however, this value still suggested some degree of clustering. Another box-plot graph was helpful in identifying the problems (Figure 1b). This plot revealed several extreme measurements, particularly at clinics B and N, which had been taken with the incorrect ruler before we corrected the pediatricians' technique but entered into the database later. Additionally, sitting height at clinic AB was now noticeably lower than the others. When questioned, the pediatrician from clinic AB explained that she had been using a sitting stool that was 40 cm high rather than the 50 cm study stool. When we added 10 cm to the sitting height measurements from site AB, the mean was consistent with the other clinics.

Further monitoring of the ICC calculations indicated improvements in data quality. In a 10/2009 download of 8,949 records, the sitting height ICC dropped from 0.28 to 0.03. A box-plot of sitting height by clinic from this download confirmed that measurements were more consistent across clinics (not shown). Even after we corrected these errors in measurement and reporting and achieved low ICC's, we found that it was important to account for clustering by center in our analyses. For example, in a preliminary analysis unadjusted for clustering by polyclinic, sitting height was significantly higher (0.49 cm, 95% CI: 0.35, 0.64) in intervention compared with control clinics. However, once we accounted for clustering using the PROC MIXED procedure within SAS, this difference disappeared (-0.09 cm, 95% CI: -0.86, 0.68).

**Figure 2:**
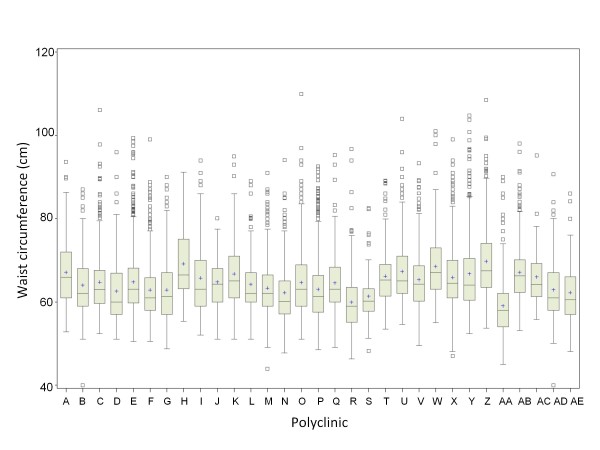
**Side-by-side box-plot of waist circumference (cm) measurements, by polyclinic**.

Monitoring ICC calculations was helpful in identifying more subtle procedural inconsistencies as well. The waist circumference ICC from the initial download was 0.10, which was higher than the ICCs for other measurements made using a measuring tape, such as hip (0.05) or head (0.03) circumference. A box-plot of waist circumference by clinic showed some variation across clinics, but the differences were less obvious than the sitting height measurements (Figure 2). We consulted the study monitors, who explained that the pediatricians were not measuring waist circumference consistently. Because the Russian translation of the manual of procedures was not entirely clear, some pediatricians were measuring waist circumference overlying the iliac crest, and others were measuring just above it. Some pediatricians also held the tape at a gentler tension than others while taking the measurement.

The ICC for systolic blood pressure (0.16) also suggested more variability across clinics than expected. A box-plot graph (not shown) of systolic blood pressure suggested that a handful of polyclinics had notably higher mean blood pressure measurements. Discussions with the pediatricians and monitors revealed that some pediatricians were not instructing the child to rest for five minutes prior to measuring blood pressure.

We also noticed an increase in the ICC for head circumference, which was relatively low until the data download in October, 2009, at which point it jumped from 0.05 to 0.13 (Table 1). Discussions with the pediatricians revealed that two clinics had been placing the measuring tape just below, as opposed to over, the occipital prominence. As a result, their measurements were lower than the other clinics. This discrepancy was not apparent until their records had been entered into the study database. Lastly, we noticed that the ICC for triceps skinfold thickness increased slightly from 0.07 to 0.11 from March to October 2009 (Table 1), but after review of the box plots and discussion with the field staff we were unable to identify any cause for this increase.

## Discussion

### Addressing and correcting procedural inconsistencies

Because we recognized most of these procedural discrepancies among clinics early in the data collection process, we were able to address them early enough to have a beneficial effect on data quality. Our first step was to provide written clarification of any ambiguity in the manual of procedures, including a description of which stool and ruler to use for sitting height, the correct placement and tension of the measuring tape for waist circumference, and the importance of five minutes rest before measuring blood pressure.

At the first refresher training workshop, we reviewed measurement procedures for sitting height, waist circumference, and blood pressure. We discussed the protocol and reinforced the correct procedures with the pediatricians in a large group session, followed by small-group practice sessions. At subsequent refresher training sessions, we revisited these topics to ensure that there was no lingering confusion.

Because some clinics had been using non-standard sitting stools, we also decided to measure the height of the stool used at each polyclinic. In cases where we confirmed that a clinic had used an incorrect ruler or study stool, we adjusted the sitting height measurements accordingly.

While the large difference in sitting height is easy to detect visually with box plots, a similar equipment error might result in a smaller absolute difference that would not be so apparent. Our experience with sitting height measurement provides a rather extreme example of differences in equipment or technique across centers that are likely very common, and may not be so readily detected if smaller in magnitude.

Following our retraining and clarification efforts at subsequent workshops, we also noticed a decrease in the ICCs for waist circumference and systolic blood pressure (Table 1). The ICC for waist circumference displayed a steady, gradual decline from a peak of 0.11 to 0.07, while the ICC for systolic blood pressure dropped from 0.18 to 0.12. However, the remaining variability was higher than we would expect, especially as we measured blood pressure in triplicate using a well calibrated machine. Our results contrast with those of Vierron et al., who found that about 20% of the variability SBP measurement with mercury sphygmomanometer and Doppler probe was attributable to differences between centers, but identified no center effect for measurements with a semiautomatic device [12]. We hypothesize that differences in the demeanor of the pediatricians and the setting of the polyclinics (e.g. the presence or absence of central heat) may explain the residual clustering we observed, but further evaluation including centralized measurement would be necessary to confirm this hypothesis.

Although we did identify the cause of the head circumference ICC increase, we were unable to clarify the procedure in time to result in a substantial decrease in the ICC value. By the time we discussed this measurement technique with the pediatricians, the two clinics that had been incorrectly measuring head circumference had completed the majority of their study visits, and we could not reliably adjust the incorrect measurements.

For measurements that are highly operator dependent, such as measurement of skinfold thicknesses and circumferences, it is likely that the reproducibility of each individual's measurements increase with experience. However, it is uncertain whether this increased precision would improve the accuracy of measurement and consistency across centers. Therefore, ongoing evaluation using ICC's, followed by retraining, is essential to ensure that measurement techniques are optimally consistent across sites.

We suggest that investigators leading similar studies do their best to ensure that data entry and reporting to study investigators occur as quickly as possible, and that auditing and retraining workshops occur as soon as possible after data collection commences, optimally within about a month. While we recognized these precepts from the start of our study, the logistical challenges of leading a large study distributed across a country with transportation challenges and limited computer accessibility outside of the central data center in Minsk were such that we were unable to entirely eliminate measurement clustering. Centralized measurement would limit the influence of measurement clustering, but it was not feasible for participants to travel to a central site for research measurements in our study, and we suspect this would be true in most other studies as well.

While an ICC of 0 might be ideal for a characteristic that truly does not vary by cluster, it is likely that many characteristics vary between centers for reasons in addition to clustered variation in measurement technique [13]. For example, there may be geographic differences in the racial or ethnic compositions of populations served in different centers, or there may be regional differences in diet or other behaviors that lead to expected variation in characteristics such as weight or BMI. These differences are a particular concern in cluster-randomized trials, in which a limited number of often heterogeneous groups often makes it difficult for randomization to distribute potential sources of confounding evenly [14]. In Belarus, the population is relatively homogenous, which is reflected by the low ICC's (< = 0.03) for many measurements such as height, weight, upper arm circumference, and bioimpedance. Although some guidelines are available for expected values of ICC, especially among studies of adults [14], little information is available for anthropometric values among children. Given the lack of regional variation in our study population, we considered an ICC to be higher than expected if it was above 0.05. However, in other settings in which regional variation is more likely, investigators will need to the appropriate threshold for investigation, perhaps based on instrument-measured characteristics or centralized measurement of selected variables.

Even after correcting errors in measurement techniques and achieving low ICC's, some clustering remains, as we found in our analysis of the intervention upon sitting height, unadjusted and accounting for clustering. Therefore, even with excellent consistency of measurement resulting in low ICC's, it is still vital to account for clustering within center in statistical analyses.

## Conclusion

While it is possible to address and correct for some measurement error through statistical analysis, proactive data monitoring is essential to ensure high-quality data collection. Early and ongoing data monitoring is especially important for multicenter studies, in which differences between centers can lead to bias and reduced study power. The goal of quality assurance efforts is procedural uniformity across centers, based on consistent and high-quality data collection techniques. As we have demonstrated here, one useful quality assurance tool is the repeated monitoring of ICCs for continuous variables during data collection; this relies on concurrent data entry. The examples presented here from the PROBIT trial show that these efforts can have a beneficial effect on data quality.

Our findings have general applicability. We demonstrated that any automation of measurement (e.g. by machines) tends to avoid clustering, but that human error can lead to a multitude of unexpected problems. We describe a simple but formal mechanism for identifying such ongoing problems during data collection. The calculation of the ICC can easily be programmed and the mechanism has wide applicability, not just to randomized controlled trials but to any study with multiple centers or with multiple observers in a single-center study.

## Competing interests

The authors declare that they have no competing interests.

## Authors' contributions

MSK, RMM, and MWG contributed to obtaining funding for this project. LBG, EO, JACS, MWG, RP, MSK, and RMM contributed to the design, analysis, interpretation, and writing or revision of the manuscript. LBG, RMM, and EO had major roles in the writing and revision of the manuscript. RP, KV, and NB made substantial contributions to the acquisition of data. All authors read and approved the final manuscript.

## Pre-publication history

The pre-publication history for this paper can be accessed here:

http://www.biomedcentral.com/1471-2288/12/29/prepub
